# Temporal decorrelation by SK channels enables efficient neural coding and perception of natural stimuli

**DOI:** 10.1038/ncomms11353

**Published:** 2016-04-18

**Authors:** Chengjie G. Huang, Zhubo D. Zhang, Maurice J. Chacron

**Affiliations:** 1 Department of Physiology, McGill University, 3655 Sir William Osler, Montreal, Quebec, Canada H3G 1Y6

## Abstract

It is commonly assumed that neural systems efficiently process natural sensory input. However, the mechanisms by which such efficient processing is achieved, and the consequences for perception and behaviour remain poorly understood. Here we show that small conductance calcium-activated potassium (SK) channels enable efficient neural processing and perception of natural stimuli. Specifically, these channels allow for the high-pass filtering of sensory input, thereby removing temporal correlations or, equivalently, whitening frequency response power. Varying the degree of adaptation through pharmacological manipulation of SK channels reduced efficiency of coding of natural stimuli, which in turn gave rise to predictable changes in behavioural responses that were no longer matched to natural stimulus statistics. Our results thus demonstrate a novel mechanism by which the nervous system can implement efficient processing and perception of natural sensory input that is likely to be shared across systems and species.

## Abstract (web summary)

The neural mechanisms underlying efficient coding of natural sensory stimuli have yet to be fully determined. Here, monitoring sensory pyramidal cells in weakly electric fish, the authors find SK channels are necessary for matching the responsiveness of neurons to natural stimuli and subsequent behavioural responses.

## Graphical abstract

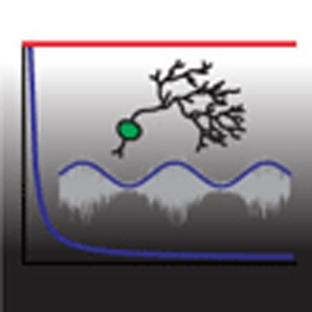

Understanding the key computations by which neurons process incoming natural sensory stimuli, thereby giving rise to perception and behaviour, remains a central problem in neuroscience. There is growing evidence that sensory systems developed coding strategies to suit a dynamic range of statistics in natural sensory stimuli^1^,^2^,^3^,^4^,^5^. Indeed, sensory systems can efficiently process input by matching their adaptation properties to natural stimulus statistics, thereby removing redundant information and thus maximizing the information transmission in presence of noise^6^,^7^,^8^. Specifically, efficient neural coding can be achieved by ensuring that the neural tuning function is inversely proportional to stimulus intensity as a function of frequency, thereby achieving a neural response that is decorrelated in the temporal domain or, equivalently, whose amplitude is independent of frequency^9^. Such ‘temporal whitening' has been observed across species and systems^10^,^11^. However, the mechanisms giving rise to efficient neural processing and, importantly, whether, and if so how, this information is decoded downstream in order to mediate perception and behaviour remains poorly understood to this day. Here we show that SK channels, which are found ubiquitously in the brain^12^, mediate efficient processing of natural stimuli by sensory neurons through temporal decorrelation and, importantly, how such processing ensures that perception is matched to natural stimulus statistics at the organismal level.

Gymnotiform wave-type weakly electric fish sense amplitude modulations (AM) of their self-generated quasi-sinusoidal electric organ discharge (EOD) through peripheral electroreceptors found on their skin. These electroreceptors in turn send afferents onto sensory pyramidal neurons within the electrosensory lateral line lobe (ELL) that subsequently project to higher brain areas, thereby mediating perception and behavioural responses^13^,^14^. Natural electrosensory stimuli have complex spatiotemporal characteristics^15^,^16^ and, as in other systems, display both first and second-order attributes that vary independently of one another and whose intensity decreases as a power law as a function of temporal frequency under natural conditions^15^,^17^. First-order stimulus attributes consist of changes in the animal's EOD amplitude caused by objects with conductivity different than that of the surrounding water (for example, prey, plants, rocks and other fish)^15^,^16^. In contrast, the second-order stimulus attributes occur exclusively during social interactions with conspecifics. For example, when two fish come into close proximity to one another, interference between both EODs gives rise to a sinusoidal stimulus (that is, a beat or first-order) whose frequency is equal to the difference between the two EOD frequencies. The beat amplitude (that is, the envelope or second-order) then depends on the relative distance and orientation between both fish and is therefore a time-varying signal under natural conditions that carries behaviourally relevant information and elicits robust behavioural responses^15^,^17^,^18^,^19^.

The responses of electrosensory neurons to first-order electrosensory stimulus attributes have been well characterized (see refs 13, 14, 20, 21 for review). Importantly, peripheral receptor afferents display high-pass filtering characteristics of time-varying first-order attributes that oppose the strongly decaying intensity as a function of temporal frequency seen under natural conditions^15^,^22^,^23^,^24^. These afferents are thus thought to efficiently process natural first-order natural electrosensory stimulus attributes by temporal whitening^15^. Each afferent trifurcates and makes synaptic contact onto pyramidal cells within three parallel maps (lateral segment, LS; centrolateral segment, CLS; centromedial segment, CMS) of the body surface within the ELL^25^. Pyramidal cells are the sole output neurons of the ELL and project to higher brain areas^26^. Parallel processing occurs at the level of the ELL as pyramidal cells within each map extract different features of first-order attributes in part through differential frequency tuning^27^,^28^,^29^ that are necessary to elicit appropriate differential behavioural responses at the organismal level^30^.

In contrast, much less is known about coding strategies used for the processing of second-order electrosensory stimulus attributes. In particular, previous studies have shown that peripheral afferents can faithfully encode these both at the single neuron and population levels^31^,^32^,^33^. However, because their tuning was found to be independent of temporal frequency, afferents do not efficiently process natural second-order electrosensory stimulus attributes through temporal whitening^31^. While previous studies have shown that ELL pyramidal cells can respond to second-order electrosensory stimulus attributes^34^, their temporal frequency tuning to these has not been investigated to date. It is therefore not known whether and, if so, how, processing of second-order electrosensory stimulus attributes by these cells is constrained by natural stimulus statistics.

We found that ELL pyramidal neurons efficiently process natural second-order electrosensory stimulus attributes through temporal whitening. Indeed, neural responses were characterized by weak correlations and by constant power for envelope frequencies spanning three orders of magnitude. Further experimentation and modelling revealed that such temporal whitening is achieved because pyramidal neurons display timescale-invariant adaptation to envelope stimuli. This adaptation enables high-pass filtering of the input through a fractional derivative operation whose exponent is matched to natural stimulus statistics. We further show that small conductance calcium-activated potassium (SK) channels mediate adaptation to envelopes in pyramidal neurons. Indeed, both pharmacological activation and inactivation of these channels altered the degree of fractional differentiation and tuning to envelope stimuli, thereby reducing efficiency of processing of natural stimuli. Importantly, these manipulations caused predictable changes in behavioural responses to natural stimuli by inducing a mismatch between behavioural sensitivity and natural stimulus statistics. Our results therefore reveal a general mechanism by which SK channels can enable efficient processing and perception of natural stimuli through scale-invariant adaptation.

## Results

### Fractional differentiation enables temporal whitening

**Figure 1:**
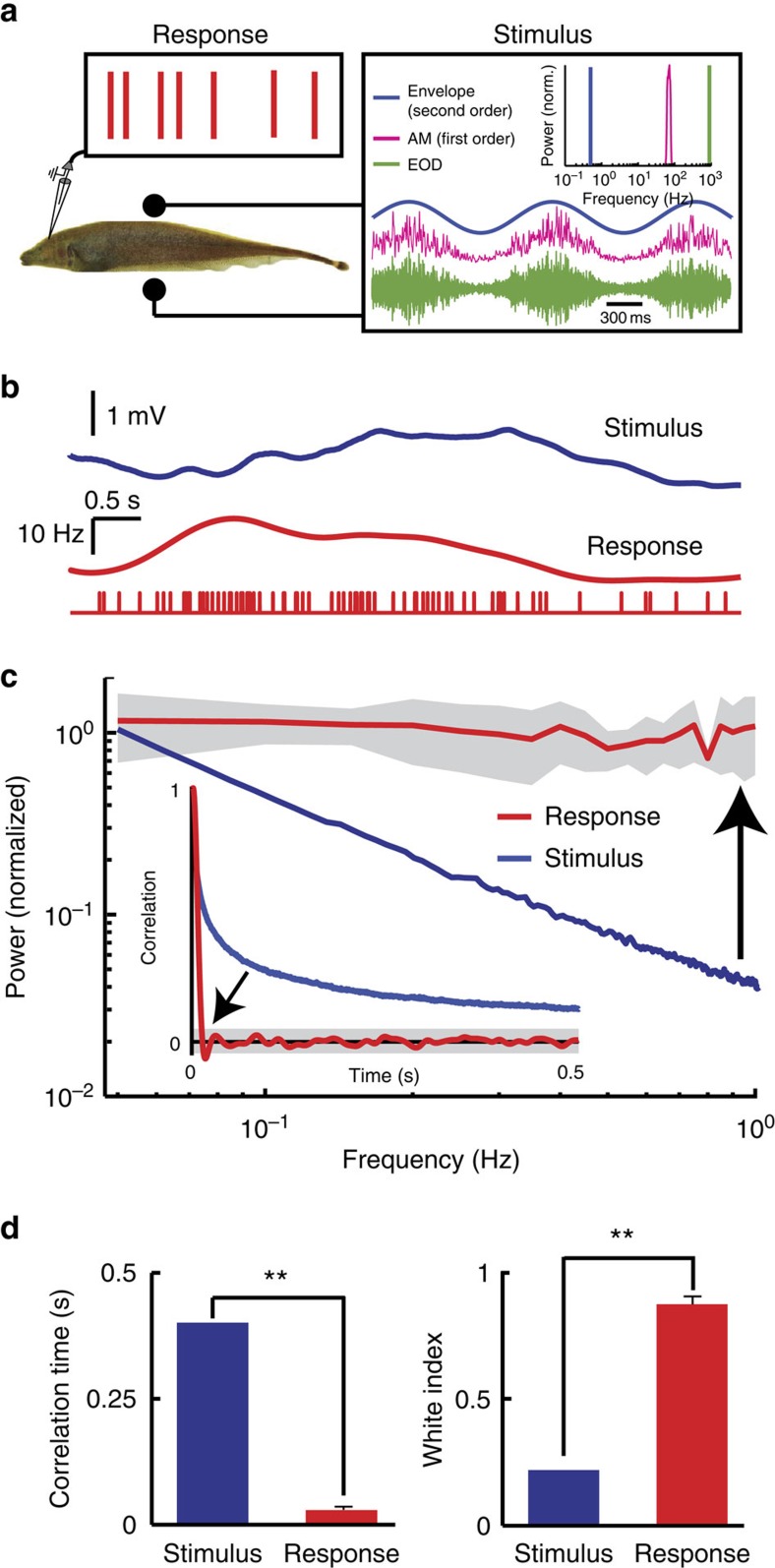
Temporal decorrelation of natural stimuli by electrosensory pyramidal neurons. (**a**) Schematic representation showing the awake behaving preparation where a stimulus is presented to the animal while neural activity is being recorded. Shown on the right are: example AM waveform (magenta), its envelope (blue), and the full signal received by the animal (green) with their respective frequency contents. (**b**) Natural envelope stimulus (blue) as well as the firing rate (middle) and spiking (bottom) response of a typical ELL pyramidal neuron. (**c**) Stimulus (blue), and population-averaged (red) neural response power spectrum. Note the flattening of the response spectrum (black arrow). The grey band shows one s.e.m. Inset: stimulus (blue), and population-averaged (red) neural response autocorrelation function. Note that the neural autocorrelation function decays to zero much faster than that of the stimulus (black arrow). The grey band shows the 95% confidence interval around zero. (**d**, left) Correlation time for the stimulus (blue) and neural response (red). (right) White index for the stimulus (blue) and neural response (red). ‘**' indicates statistical significance at the *P*=0.01 level using a Wilcoxon rank-sum test with *N*=14.

We recorded ELL pyramidal neuron responses to stimuli (*n*=14) in awake and behaving animals (Fig. 1a). Our stimuli consisted of a fast time-varying waveform (first-order) with a slow time-varying amplitude (that is, the envelope or second-order) as encountered under natural conditions^15^,^18^. Figure 1a shows an example AM waveform (magenta), its envelope (blue), as well as the full signal received by the animal (green) with respective frequency content. It is important to realize that the animal's unmodulated EOD is a carrier and that the meaningful stimulus here is the EOD AM. Thus, we note that the first- and second-order features of the stimulus actually correspond to the second- and third-order features of the full signal received by the animal, respectively.

We considered envelope waveforms that either varied sinusoidally or whose timecourse mimicked of that seen under natural conditions (Fig. 1b, see Methods). Specifically, for the latter case, the envelope autocorrelation decayed over a time window of 400 ms (Fig. 1c, inset) while the envelope power decayed as a power law with exponent *α*_stim_=−0.8 (Fig. 1c). We found that pyramidal neurons displayed robust responses to such stimuli (Fig. 1b, bottom). Interestingly, further analysis revealed that pyramidal neurons perform temporal decorrelation of natural envelope stimuli. Indeed, the response autocorrelation function decayed to zero much faster than that of the stimulus over a time window of 27.5 ms (Fig. 1c, inset) as quantified by significant differences in correlation time (see Methods, Fig. 1d, left). Moreover, the response power spectrum was constant for frequencies spanning three orders of magnitude (Fig. 1c), indicating whitening. Indeed, the population-averaged neural whitening index was significantly larger than that of the stimulus (Fig. 1d, right). We note that ELL pyramidal cells can be classified as either ON or OFF-type based on whether they respond with increases or decreases in firing rate to increases in EOD AM (that is, first-order), respectively^35^. Cells in our data set could be easily identified as either ON or OFF-type based on responses to sinusoidal AMs (Supplementary Figs 1A,B). We however found no significant differences between ON and OFF-type pyramidal cell responses to envelope stimuli (Supplementary Figs 1C,D). Data from each cell class were thus pooled in subsequent analyses.

**Figure 2:**
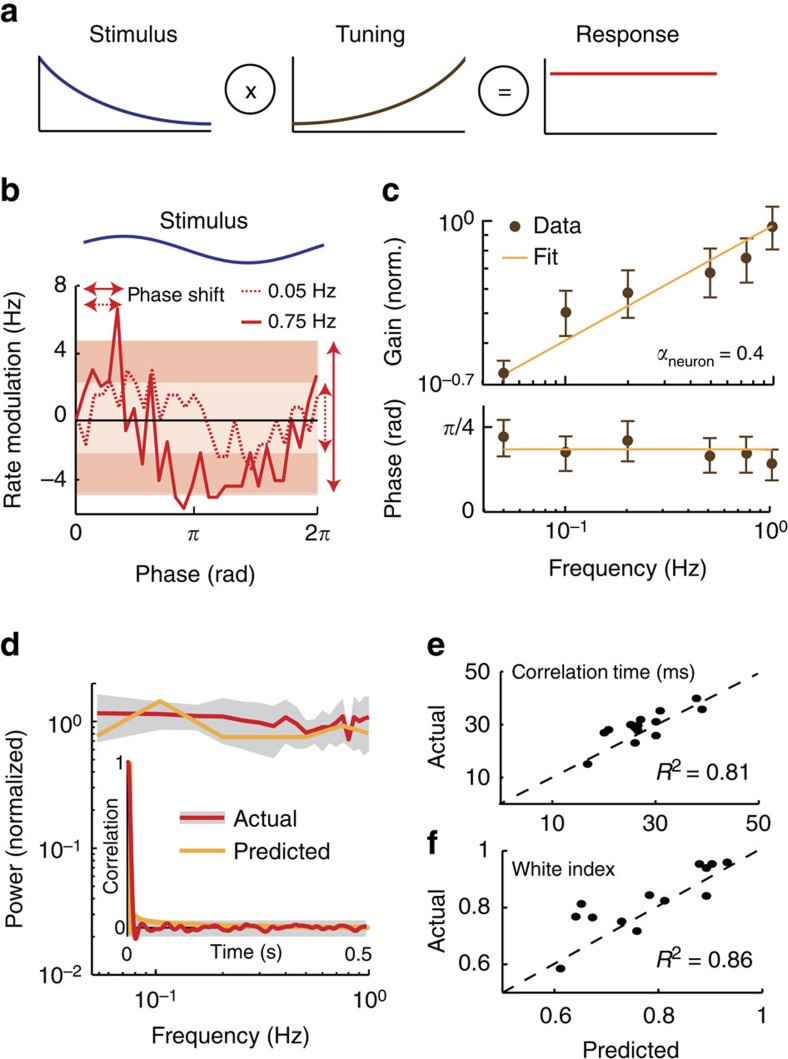
Fractional differentiation by electrosensory pyramidal neurons achieves temporal decorrelation. (**a**) Schematic representation showing that the neural tuning function (middle) must oppose the decay in the stimulus power (left) in order to achieve a neural response that is constant (right). (**b**) Phase histograms showing the firing rate modulation in response to the stimulus (blue) for low (dashed red) and high (solid red) envelope frequencies. The bands and vertical arrows show the amplitudes of the best sinusoidal fits (not shown for clarity) for both frequencies, which are used to compute gain. The horizontal arrows show the phase shift between the stimulus and the firing rate modulation signal. (**c**) Population-averaged (brown) sensitivity (top) and phase (bottom) obtained from sinusoidal stimuli (*N*=14). The solid orange lines show the gain and phase of the best-fit fractional derivative. (**d**) Predicted (orange) and actual (red) response power spectra to natural stimuli (*N*=14). The grey band shows 1 s.e.m. Inset: predicted (orange) and actual (red) response autocorrelation function. The grey band shows the 95% confidence interval around zero. (**e**,**f**) Predicted as a function of actual correlation time and white index, respectively.

How is temporal whitening of natural stimuli by pyramidal neurons achieved? Theory posits that such whitening is achieved by ensuring that the neuron's tuning curve is matched to the statistics of natural input^9^. Neural sensitivity should then be highest for frequencies at which stimulus power is lowest. A simple derivation (see Methods) predicts that, in order to achieve temporal whitening of stimuli whose power decreases with exponent *α*_stim_= −0.8, neural sensitivity should increase as a power law with exponent *α*_neuron_=−*α*_stim_/2=0.4 (Fig. 2a).

To verify this prediction, we recorded pyramidal neuron responses (*n*=14) to sinusoidal envelope stimuli with frequencies spanning the behaviourally relevant range (0.05–1 Hz). We found that pyramidal neurons responded to such stimuli through sinusoidal modulations in firing rate that increased in amplitude as a function of frequency (Fig. 2b). We then used linear systems identification and plotted the sensitivity and phase relationships between stimulus and neural response as a function of frequency (Fig. 2c). Our results show that sensitivity indeed increased as a power law as a function of frequency with exponent 0.4 (Fig. 2c, top), while the phase remained constant (Fig. 2c, bottom). Such phase constancy is typical of fractional differentiation, a mathematical operation that is thought to be advantageous for coding^36^. Fractional differentiation in the time domain is equivalent to linearly filtering by a transfer function with gain (*2πf*)^α^ and phase *απ/2* (see Methods), where *f* is the frequency and *α* is the order of differentiation. We thus fitted a fractional derivative model with *α=*0.4 to our data (see Methods) and found an excellent fit (Fig. 2c). Importantly, this simple model correctly predicted temporal decorrelation and whitening seen in response to naturalistic envelope stimuli (Fig. 2d) as quantified by both correlation time (Fig. 2e) and whitening index (Fig. 2f). We conclude that temporal whitening of natural envelopes occurs because pyramidal neurons high-pass filter the input stimulus through fractional differentiation whose exponent is precisely matched to natural stimulus statistics.

### A simple model reproduces experimental data

**Figure 3:**
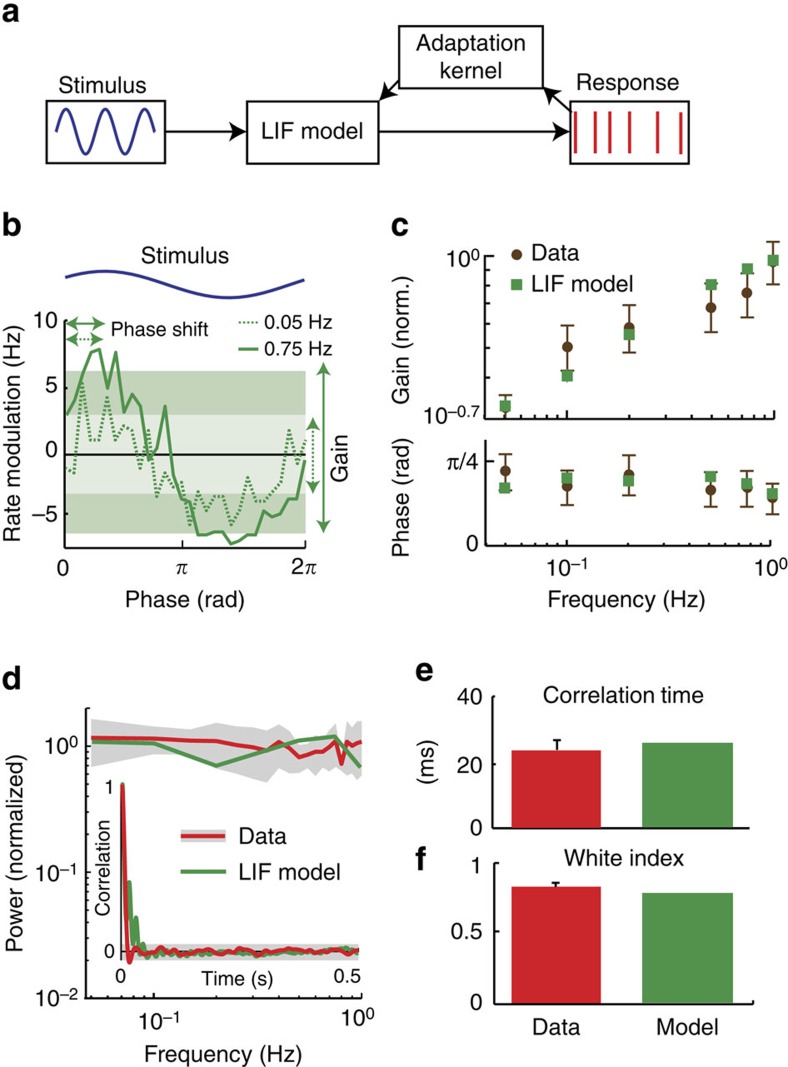
A simple model with power law adaptation implements temporal decorrelation by fractional differentiation. (**a**) Model schematic representation in which the stimulus (blue) is fed to a leaky integrate-and-fire (LIF) neuron model with an adaptation kernel that decays as a power law as a function of time. The spiking output of the model (red) was analysed in the same manner as the experimental data. (**b**) Phase histograms showing the firing rate modulation in response to the stimulus (blue) for low (dashed green) and high (solid green) envelope frequencies. The bands and vertical arrows show the amplitudes of the best sinusoidal fits (not shown for clarity) for both frequencies, which are used to compute gain. The horizontal arrows show the phase shift between the stimulus and the firing rate modulation signal. (**c**) Population-averaged sensitivity (top) and phase (bottom) for the data (brown) and our LIF model (green) obtained for sinusoidal stimuli. (**d**) Response power spectra to natural stimulation for our experimental data (red) and LIF model (green). The grey band shows 1 s.e.m. for the experimental data. Inset: response autocorrelation function to natural stimulation for our experimental data (red) and LIF model (green). The grey band shows the 95% confidence interval around zero for the experimental data. (**e**,**f**) Population-averaged values obtained from experimental data (red) and for our LIF model (green) for correlation time and white index, respectively.

To gain insight into the mechanism which enables pyramidal neurons to efficiently process natural stimuli through fractional differentiation, we built a simple model based on the leaky integrate-and-fire formalism that included a spike-activated adaptation current that decayed as a power law in the absence of firing^37^, see Methods (Fig. 3a). The output model spike train was analysed in the same way as our experimental data. Numerical simulation revealed that this simple model accurately reproduced our experimental data (compare Figs 2b and 3b). Indeed, the model neuron's sensitivity and phase closely matched those obtained experimentally (Fig. 3c). Importantly, the model also accurately reproduced temporal whitening in response to naturalistic stimuli (Fig. 3d) as quantified by correlation time (Fig. 3e) and white index (Fig. 3f).

**Figure 4:**
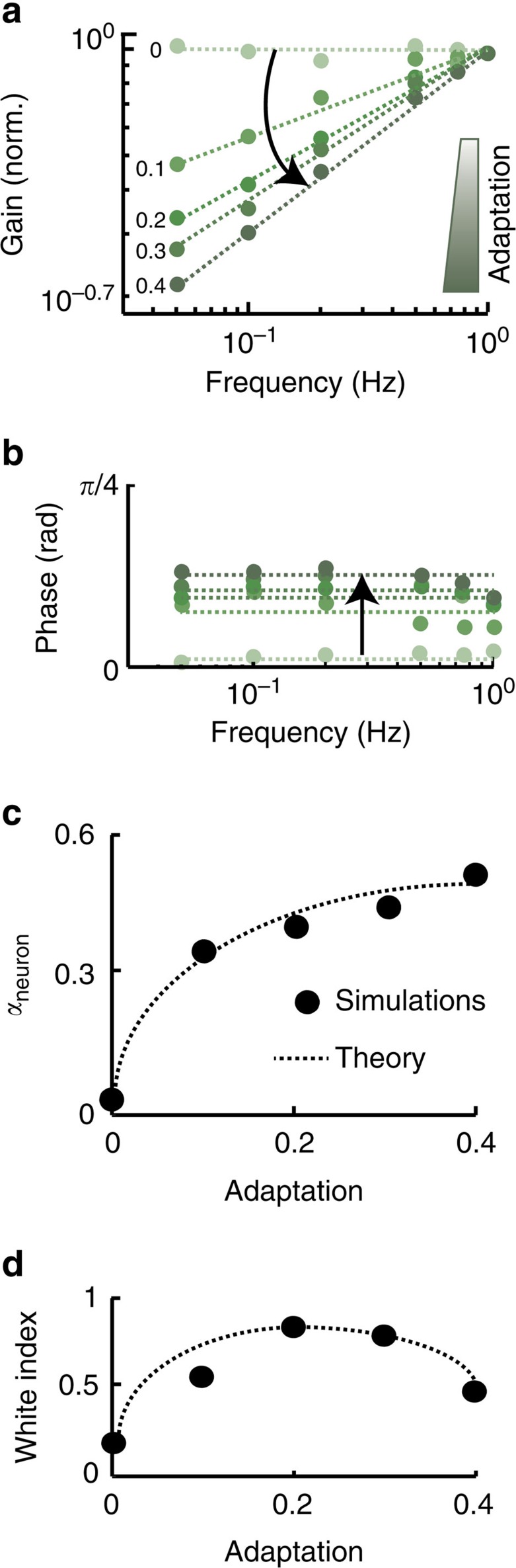
Our model predicts that adaptation strength is critical to ensure efficient processing of natural stimuli. (**a**,**b**) Model gain and phase as a function of frequency for different amounts of adaptation, respectively. For each amount of adaptation, the circles show the values obtained from numerical simulation and the dashed lines those from the best-fit fractional derivative model. Note the progressive steepening of the gain curve as well as the increase in phase as adaptation is increased (black arrows). (**c**) Neural exponent *α*_neuron_ as a function of adaptation showing values obtained from numerical simulation (black circles) and theoretical prediction (dashed line). (**d**) White index computed in response to a natural stimulus with exponent *α*_stim_=−0.8 as a function of adaptation showing values obtained from numerical simulation (black circles) and theoretical prediction (dashed line).

To understand how adaptation can lead to efficient processing of natural stimuli, we next systematically varied the strength of the adaptation current in our model. We found that, without adaptation, our model displayed constant sensitivity and no phase lead in response to envelope stimuli (light green curves in Fig. 4a,b). Increasing the adaptation strength led to sensitivity curves which increased more steeply as a function of frequency and furthermore increased phase lead (compare light and dark green curves Fig. 4a,b), consistent with increases in the neural exponent *α*_neuron_ (Fig. 4c). These results have important implications as they predict that, for a given adaptation strength, our model can only achieve temporal decorrelation/whitening of stimuli whose power decays with a given exponent. This was verified by plotting the whitening index for naturalistic envelope stimuli (that is, *α*_stim_=−0.8) as a function of the adaptation strength. Indeed, both lower and higher adaptation strength led to tuning curves that were not matched to natural stimulus statistics and lowered coding efficiency as quantified by lower white index values (Fig. 4d).

Our model therefore makes two important predictions. The first is that, in order to observe temporal whitening of scale-invariant natural stimuli through fractional differentiation, neurons must display adaptation that is also scale invariant (that is, decay as a power law). The second is that temporal whitening is only achieved for a given adaptation strength. Thus, increases or decreases in the adaptation strength will alter neural tuning and lead to sub-optimal processing of natural stimuli.

### Pyramidal neurons display power law adaptation

**Figure 5:**
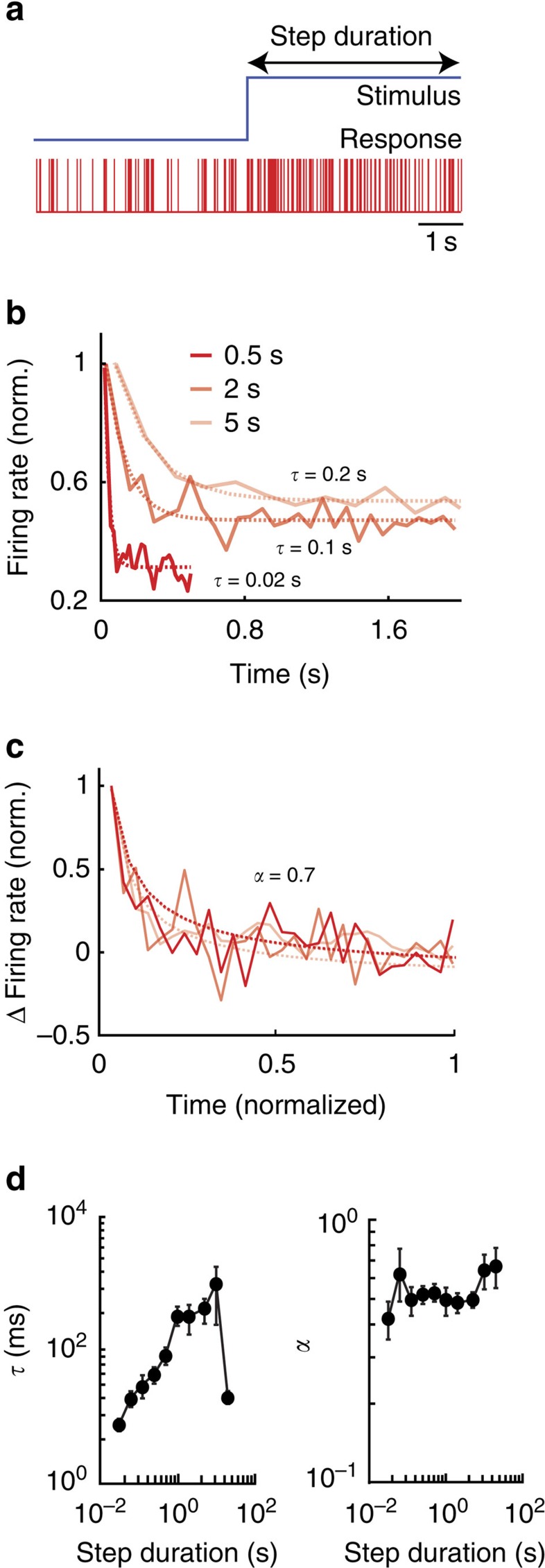
Electrosensory pyramidal neurons display power law adaptation in response to step changes in envelopes. (**a**, top) Step stimulus that switches from a low to a high value (onset) with duration indicated by the black arrow. (bottom) Spiking response from a typical electrosensory pyramidal neuron to this stimulus. (**b**) Time-dependent firing rate following the step onset (solid red) for three different step durations and corresponding best exponential fits (dashed red). The numbers give the time constants of these fits: note the different values obtained for different step durations. We note that firing rate normalization does not affect the value of the fitted exponential time constants. (**c**) Normalized change in firing rate as a function of normalized time following the step onset (solid red) for the same three different step durations and corresponding power law fits (dashed red). Note that the curves now superimpose and are thus well fit by power laws with similar exponents. (**d**, left) Population-averaged exponential time constant *τ* as a function of step duration. (right) Population-averaged power law exponent *α* as a function of step duration (*N*=23).

To test whether pyramidal neurons display scale-invariant adaptation, we recorded their responses to step changes in envelope (Fig. 5a). We found that pyramidal neurons responded to such stimuli by a rapid increase in firing rate followed by a slower decay following the step onset, which is characteristic of spike frequency adaptation (Fig. 5a). If adaptation displays a characteristic timescale (that is, is not scale invariant), then we expect that the peristimulus time histogram (PSTH) responses to step onset with different duration will all be well-fit by an exponential curve with the same time constant, whereas a power law will instead give a poor fit. If adaptation is instead scale invariant, then we expect that PSTH responses to step onset with different duration will all be well fit by a power law curve with the same exponent. The apparent decay time constant of adaptation as quantified by fitting an exponential will then be inversely proportional to the step duration^6^,^38^.

To test our hypothesis, we plotted the time-dependent firing rate in response to steps with different durations. The curves obtained did not overlap and were each well fit by exponentials but with different time constants (Fig. 5b). Rescaling both the firing rate and time led to strong overlap between the curves that were all well fit by power laws with the same exponent (Fig. 5c). We note that rescaling both firing rate and time will not alter the power law exponent. Thus, our results suggest that the timecourse of adaptation in ELL pyramidal cells follows a power law rather than an exponential. We next systematically varied the step duration and found that, while the exponential time constant varied strongly as a function of step duration (Fig. 5d, left), the power law exponent was instead relatively independent of step duration (Fig. 5d, right). We conclude that pyramidal neurons indeed display scale invariant (that is, power law) adaptation in response to envelopes as predicted by our model.

### SK channels promote efficient coding of natural stimuli

So far, we have shown that ELL pyramidal neurons can efficiently process natural stimuli through temporal decorrelation because of fractional differentiation, which ensures that the neural tuning increases as a power law with exponent *α*_neuron_ that is precisely related to the power law exponent of the stimulus *α*_stim_. Our model predicted that such fractional differentiation can be explained by including an adaptation current whose timecourse follows a power law which was confirmed experimentally. Importantly, our model also predicted that changing the level of adaptation can strongly affect *α*_neuron_, which should decrease coding efficiency. Thus, we next tested experimentally whether modifying adaptation in pyramidal neurons will alter their tuning exponent *α*_neuron_, and whether this will decrease coding efficiency as quantified by the white index.

**Figure 6:**
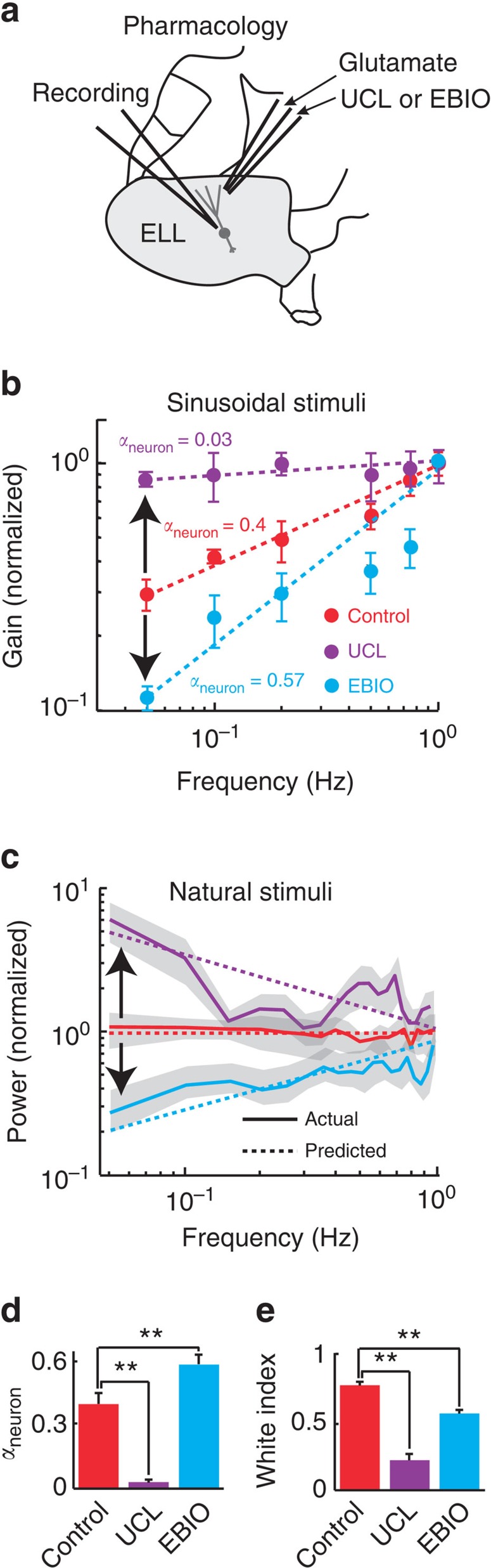
Pharmacological inactivation and activation of SK channels alter neural sensitivity and both reduce coding efficiency of natural stimuli. (**a**) Schematic representation showing how a double-barrel electrode approaches and can eject glutamate as well as either UCL (SK channel antagonist) or EBIO (SK channel agonist) in the near vicinity of the pyramidal neuron being recorded from. (**b**) Normalized gain as a function of frequency obtained for sinusoidal stimuli under control (red), after UCL application (purple) and after EBIO application (cyan). The circles show the experimental data and the dashed lines the best power law fits with exponents *α*_neuron_ given in the figure. UCL and EBIO application decreased and increased the steepness of the curve, respectively (black arrows). (**c**) Response power spectra to natural stimuli under control (solid red), after UCL application (solid purple) and after EBIO application (solid cyan). The dashed lines show the predicted values obtained from the power law fits in **b**. UCL and EBIO application led to response power spectra that were no longer independent of frequency (black arrows). (**d**) Population-averaged neural exponent *α*_neuron_ under control (red), after UCL application (purple) (*N*=6), and after EBIO application (cyan) (*N*=8). (**e**) Population-averaged white index values under control (red), after UCL application (purple) (*N*=6), and after EBIO application (cyan) (*N*=8). ‘**' indicates statistical significance at the *P*=0.01 level using a one-way ANOVA with *post hoc* Bonferroni correction.

We focused on small conductance calcium-activated potassium (SK) channels. This is because previous results have shown that pharmacologically activating and inactivating these currents will increase and decrease adaptation in ELL pyramidal neurons, respectively^39^,^40^. We thus hypothesized that pharmacological activation and inactivation of SK channels will increase and decrease fractional differentiation by pyramidal neurons, respectively, thereby altering tuning. Both manipulations are then predicted to decrease efficient coding of natural stimuli by temporal whitening. We thus micro-injected the SK channel antagonist UCL-1684 (UCL) as well as the SK channel agonist 1-EBIO (EBIO) in the ELL using well-established methodology (Bastian^41^; Deemyad *et al.*^42^; Supplementary Fig. 2A, see Methods) (Fig. 6a). We note that previous studies have shown that injection of saline alone using this methodology does not alter pyramidal neuron activity^41^,^42^. Consistent with previous results^39^,^43^, we found that UCL and EBIO application both strongly altered pyramidal neuron activity in the absence of stimulation (Supplementary Figs 2B–D).

If our hypothesis is true, then we expect that UCL application will decrease the neural tuning exponent *α*_neuron_ as neural sensitivity should then increase less steeply as a function of frequency when using sinusoidal stimuli. In contrast, we expect that EBIO application will increase the neural tuning exponent *α*_neuron_ as neural sensitivity should then increase more steeply as a function of frequency. Consistent with these predictions, neural sensitivity indeed became relatively independent of frequency following UCL application as quantified by a decrease in *α*_neuron_ (Fig. 6b, compare red and purple). Neural sensitivity increased more steeply as a function of frequency after EBIO application as quantified by an increase in *α*_neuron_ (Fig. 6b, compare red and cyan).

We next tested whether changes in neural tuning do indeed decrease coding efficiency when instead using natural stimuli. To do so, we next plotted the response power spectra before and after application of either UCL or EBIO. We found that, after UCL application, the response power spectrum decayed as a function of frequency (Fig. 6c, compare red and purple). In contrast, the response power spectrum increased as a function of frequency after EBIO application (Fig. 6c, compare red and cyan). The changes in power spectra observed were in agreement with predictions from our simple model (Fig. 6c, compare dashed and solid curves) that were based solely on the changes in *α*_neuron_ (Fig. 6d). Importantly, confirming our prediction; UCL and EBIO application both significantly reduced coding efficiency as quantified by the white index (Fig. 6e).

### SK channels in ELL determine behavioural responses

**Figure 7:**
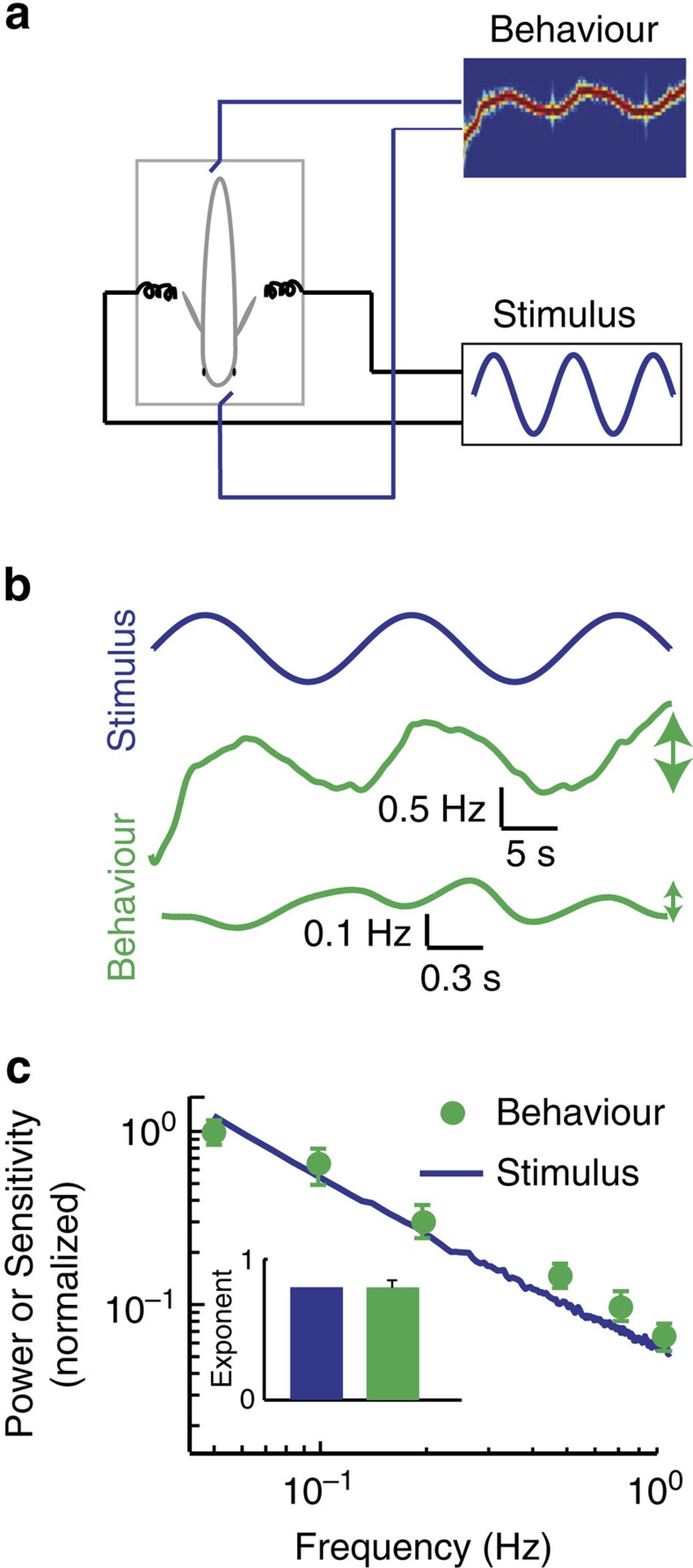
Weakly electric fish display behavioral responses that are matched to natural stimulus statistics. (**a**) Schematic representation showing the behavioural setup in which the animal's behavioural responses to stimuli are recorded by continuously monitoring its EOD, whose spectrogram indicates the time-varying frequency. (**b**) Stimulus (blue) and time-varying EOD frequency responses (green) to 0.05 Hz (middle) and 0.75 Hz (bottom) sinusoidal stimuli. Note the smaller changes in EOD frequency in response to the 0.75 Hz stimulus (vertical green arrows). (**c**) Behavioural response sensitivity (green) is matched to the power spectrum (blue) of natural envelope stimuli. Inset: population-averaged power-law exponents from behavioural sensitivity (green) and from natural envelope stimuli (blue).

Information transmitted by neurons is only useful to an organism if it is actually decoded downstream. Thus, we next investigated how efficient coding of natural stimuli by ELL pyramidal neurons mediates perception. To do so, we took advantage of the fact that weakly electric fish display robust behavioural responses to envelope stimuli^18^,^31^ (Fig. 7a). These consist of changes in the animal's EOD frequency that follows the stimulus' detailed timecourse but whose magnitude decreases with increasing frequency (Fig. 7b). Behavioural response sensitivity is matched to natural stimulus power (Fig. 7c). Indeed, both curves decreased as a power law with exponents *α*_behaviour_ and *α*_stim_ that were not significantly different from one another (Fig. 7c, inset). This matching ensures that behavioural sensitivity is greatest for stimulus frequencies that tend to occur most frequently in the natural environment^4^,^18^.

**Figure 8:**
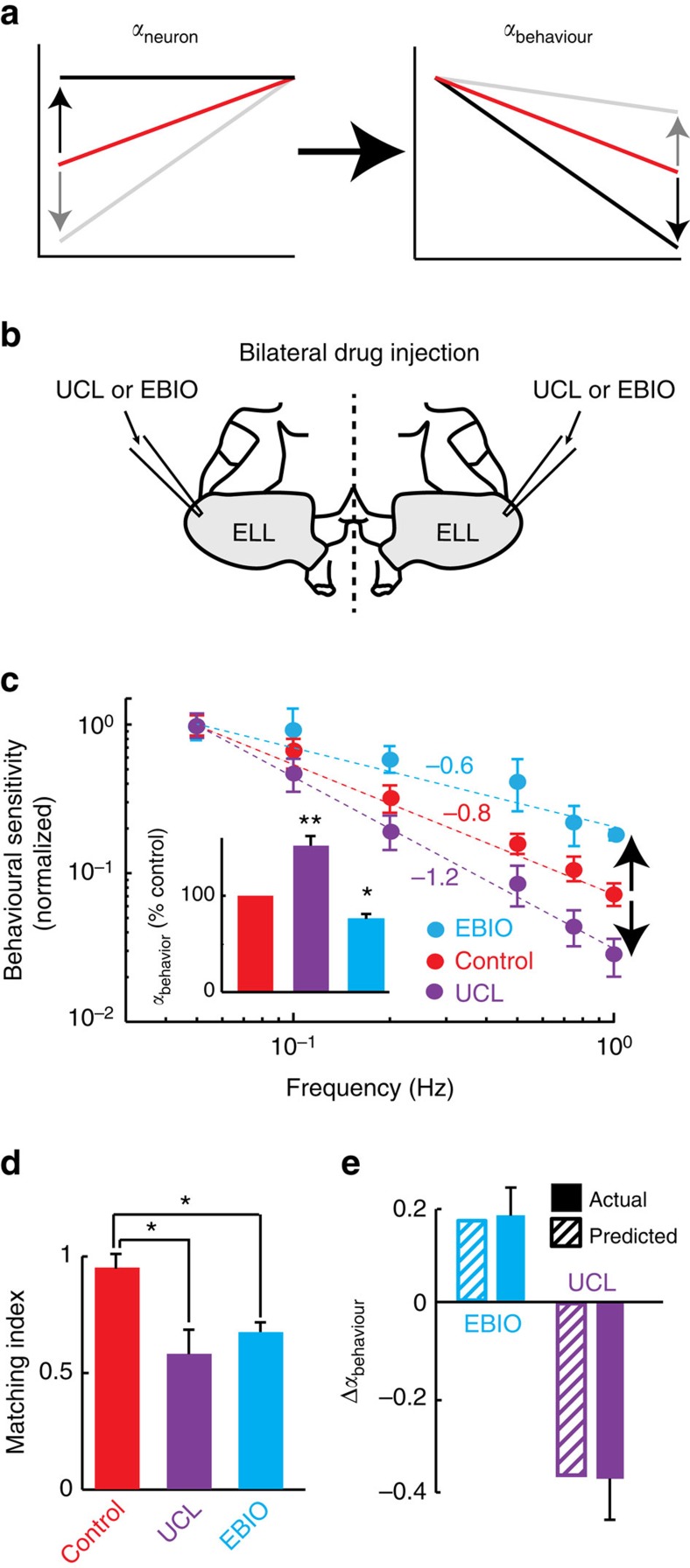
Changes in neural sensitivity caused by pharmacologically manipulating SK channels cause predictable changes in behavioural responses. (**a**) Schematic representation showing how changes in the neural tuning characterized by exponent *α*_neuron_ are predicted to cause changes in behavioural sensitivity characterized by exponent *α*_behaviour_. (**b**) Schematic representation of the bilateral ELL drug injection setup by which UCL or EBIO is injected simultaneously in both ELL's on each side of the brain via two electrodes. (**c**) Population-averaged normalized behavioural sensitivities under control (red), after UCL application (purple) and after EBIO application (cyan). The circles show the experimental data and the dashed lines the best power law fits with exponents *α*_behaviour_ given in the figure. Inset: population-averaged *α*_behaviour_ values under control (red), after UCL application (purple) and after EBIO application (cyan). (**d**) Population-averaged matching index between behavioural response and natural stimulus statistics under control (red), after UCL application (purple) (*N*=6), and after EBIO application (cyan) (*N*=6). Both drugs significantly decreased the matching index value. (**e**) Actual (solid) and predicted (striped) changes in exponent *α*_behaviour_ caused by UCL (purple) and EBIO (cyan) application. The changes were predicted solely from the changes in neural tuning exponent *α*_neuron_ shown in Fig. 6d. ‘**' and ‘*' indicate statistical significance a using a one-way ANOVA with *post hoc* Bonferroni correction at the *P*=0.01 and 0.05 levels, respectively.

We hypothesized that behavioural sensitivity is directly related to ELL pyramidal neuron tuning. Thus, changing the neural tuning exponent *α*_neuron_ should cause changes in the behavioural exponent *α*_behaviour_ (Fig. 8a) and a simple model predicts that Δ*α*_behaviour_=−Δ*α*_neuron_ (see Methods). To test our hypothesis, we injected UCL and EBIO bilaterally into the ELL (Fig. 8b)^42^,^44^ (see Methods). As a control, injection of saline alone had no significant effect on behavioural responses (Supplementary Fig. 3). In contrast, UCL and EBIO injection both strongly altered behavioural sensitivity (Fig. 8c). Indeed, behavioural sensitivity decreased more steeply following UCL application as quantified by a greater behavioural exponent *α*_behaviour_ (Fig. 8c, compare red and purple, Fig. 8c, inset). In contrast, behavioural sensitivity decreased less steeply after EBIO application as quantified by a lesser behavioural exponent *α*_behaviour_ (Fig. 8c, compare red and cyan, Fig. 8c, inset). Importantly, behavioural sensitivity was no longer matched to natural stimulus statistics after both UCL and EBIO application (Fig. 8d). Consistent with our simple model, changes in behavioural tuning *α*_behaviour_ following the UCL and the EBIO applications were consistent with predictions made from changes in *α*_neuron_ (Fig. 8e). Thus, we conclude that efficient processing of natural envelope stimuli by ELL pyramidal neurons does indeed ensure that behavioural sensitivity at the organismal level is matched to natural stimulus statistics.

## Discussion

Envelopes constitute a critical component of the natural electrosensory environment as they carry information about the relative positions between conspecifics as well as their identities^15^,^17^. In particular, envelopes can arise during movement between two conspecifics as well as from the static interactions between the electric fields of three of more fish. While the former movement envelopes generally tend to contain low (<1 Hz) temporal frequencies^15^,^17^,^18^, the latter ‘social' envelopes tend to instead contain higher (>1 Hz) temporal frequencies^15^,^17^. Behavioural studies have shown that weakly electric fish can perceive both categories of envelopes^18^,^19^. While it is known that electrosensory neurons respond to mimics of social envelope stimuli^32^,^34^,^45^, little is known about the coding of movement envelope stimuli.

Here we have shown that ELL pyramidal neurons receiving direct synaptic input from peripheral afferents optimally process natural movement envelope stimuli because of scale-invariant adaptation. Such adaptation leads to high-pass filtering of envelopes through fractional differentiation whose exponent is matched to natural stimulus statistics, thereby removing temporal correlations in the response or, equivalently, whitening the response power across frequencies. By whitening the response power across frequencies, the brain should be able to encode the most important information in natural sensory stimuli while discarding any redundancies, most often found in the high-power, low-frequencies range. This agrees with efficient coding theory, which states that optimality is achieved by adapting to the natural stimulus statistics, and by completely removing any correlations which are potentially present in the signals to be encoded^46^. This process was shown to critically depend on SK channels. It was previously shown that SK2 channels are located on the somata of ON-type pyramidal neurons, while SK1 channels are instead located on the apical dendrites of both OFF and ON-type pyramidal neurons^47^. Despite these differences, even when we segregated pyramidal neurons into ON and OFF types, the temporal whitening of natural second-order stimulus statistics did not differ significantly. Furthermore, when we applied the SK channel antagonist and agonist in the apical dendritic tree, we observed that each of their effects were similar in ON and OFF-type pyramidal neurons. We therefore hypothesize that SK1 channels are sufficient to give rise to optimized envelope processing and perception. Pyramidal neurons receive large amounts of feedback on their apical dendrites^48^ that help refine responses to electrosensory stimuli^49^,^50^,^51^ and previous studies have shown that pharmacological inactivation of SK1 channels strongly disrupted responses to first-order electrosensory stimuli^43^. It is therefore likely that SK1 channels optimize processing of movement envelope stimuli by altering feedback input to ELL pyramidal neurons but further studies are needed to gain more understanding of the underlying mechanisms. We also note that, while our results make it clear that disrupting pyramidal neuron responses to envelopes leads to predictable changes in behaviour, further studies are needed to understand how downstream targets of pyramidal neurons will respond to this behaviourally relevant stimulus feature.

Our results suggest a novel mechanism by which neural responses can be adaptively optimized to process natural stimuli. Indeed, our modelling and pharmacological manipulations suggest that SK channel conductance is critical for optimizing processing of natural stimuli with given statistics. If true, then regulating SK channel conductances could serve as a dynamic control for adaptive optimized processing of stimuli following changes in the environment. In particular, we predict that exposing the animals to envelope stimuli whose power law exponents differ from those seen in the natural environment will give rise to changes in SK channel conductance, thereby altering ELL pyramidal neuron tuning in order to optimize processing of these new stimuli through temporal decorrelation/whitening, thus altering and optimizing perception and behaviour. Dynamic regulation of SK channel conductance could come from serotonergic modulation as previous studies have shown that elevating serotonin levels inhibits SK channels in ELL pyramidal neurons^40^,^42^. Finally, it should be noted that our simplistic model predicts a direct link between the ELL pyramidal neurons and behaviour. These behavioural responses are likely to result from further processing of ELL by several downstream areas possibly including forebrain. In this context, the observed match between changes in ELL neural and behavioural responses induced by pharmacologically manipulating SK might thus appear surprising. This match should not, however, be taken as evidence that downstream brain areas always merely relay information carried in ELL pyramidal cell spike trains. Rather, it is likely that these are involved in other aspects of behavioural responses to envelopes that were not considered in the current study such as the previously described habituation to repeated presentations of the same envelope stimulus^18^. Further studies are needed to test these interesting hypotheses to demonstrate how processing and perception of natural stimuli are dynamically optimized based on input statistics, but are clearly beyond the scope of this paper.

We note that our results showing that the electrosensory system efficiently process second-order natural electrosensory stimulus attributes in no way imply that other stimulus attributes (for example, first-order) are not also processed efficiently. This is because previous studies have shown that both first- and second-order attributes are processed in parallel by different subset of neurons in higher order areas^34^. However, both attributes must first be processed by the same neurons in more peripheral areas before reaching these. In particular, peripheral receptor afferents respond to both first- and second-order electrosensory stimulus attributes, but display differential frequency tuning to each attribute. Indeed, while afferents are preferentially tuned to higher temporal frequencies for first-order attributes^22^,^23^,^24^, their tuning to second-order attributes is instead independent of temporal frequency^31^. For first-order statistics, the power law exponent characterizing the rate at which sensitivity increases is matched to the power law exponent characterizing the rate at which stimulus power decays as a function of frequency; afferents are thus thought to efficiently encode the first-order natural electrosensory stimulus attributes through temporal whitening^15^. However, no such match was observed for second-order attributes as the sensitivity does not increase as a function of temporal frequency in order to oppose the rate at which envelope power decays as a function of frequency^31^. Thus, peripheral afferents do not efficiently process natural second-order electrosensory stimulus attributes through temporal whitening.

Our results show that efficient processing instead emerges at the level of the ELL and requires SK channels. It is important to note here that we only recorded from pyramidal cells within LS, which displays the greatest SK channel expression^39^. Since pyramidal cells within CLS and CMS display considerably less expression, we predict that these will not efficiently process natural second-order electrosensory stimulus attributes through temporal whitening. This is not a problem as pyramidal cells within CLS and CMS have been shown to be involved in the processing of other stimulus attributes^30^,^52^. These include those encountered during prey capture. Indeed, weakly electric fish display robust behavioural responses showing that they can reliably and accurately detect the presence of the underlying weak stimuli as they then execute a series of movements to capture the prey^16^. Such behaviour is likely to require multisensory integration as the animal then experiences simultaneous stimulation of its active electrosensory, passive electrosensory and lateral line systems^53^. In particular, the passive electric sense is likely to make a substantial contribution to allow the animal to first successfully detect the presence of a prey as ampullary electroreceptors are exquisitely sensitive to the resulting small-amplitude exogenous electric fields^15^. The perturbations of the animal's own electric field caused by the prey during the detection phase are very weak and will in turn cause very small perturbations in the activities of tuberous electroreceptors^16^. While these can theoretically be decoded^54^, further studies are needed to understand whether and, if so, how neural circuits of the active electric sense actually decode these faint signals in the presence of substantial variability. It is thought that the active electric sense makes an important contribution to give the animal sensory feedback as to the prey's location as it is executing a series of movements to bring the prey close to its mouth. ELL pyramidal cells within CLS and CMS are then likely to be involved as both their frequency tuning^27^,^28^,^52^ and receptive field organization^55^,^56^ are optimized to the statistics of the input. Importantly, we note that LS pyramidal cells, which were the focus of the current study, do not solely process second-order electrosensory stimulus attributes. Indeed, previous results have shown that these cells respond to natural communication calls consisting of high-frequency transients^29^. Since SK channels are major determinants of frequency tuning in LS pyramidal cells^39^,^57^, it is likely that these will also contribute to shaping responses to natural communication stimuli. It is then conceivable that SK channel expression would be not only constrained to optimally process second-order electrosensory stimulus attributes as shown here but might also be constrained to optimally process natural communication stimuli as well.

Thus, it is likely that electrosensory-coding strategies are constrained to efficiently process natural stimuli. However, these will differ depending on the subset of natural stimuli considered and are likely to involve multiple sensory modalities. A complete understanding of these will require further studies and is clearly beyond the scope of this paper that only considered second-order electrosensory stimulus attributes.

It is very likely that our results will be applicable to other systems. First, we note that SK channels found in weakly electric fish display ∼86% sequence identity with those found in mammals^39^. SK channels are furthermore expressed ubiquitously in the brain and are key determinants of spike frequency adaptation^12^. Second, natural stimuli have been shown to also exhibit power spectra that decay as a power law in the visual^5^,^58^ and auditory^59^ systems and also display first- and second-order attributes. Third, growing evidence suggests that neural coding strategies are adapted to natural stimulus statistics by optimizing neural responses via temporal decorrelation/whitening across systems and species^10^,^11^. In particular, adaptation to second-order stimulus attributes is widely observed^7^,^8^,^60^. Further, our proposed mechanisms underlying temporal decorrelation/whitening, namely high-pass filtering by fractional differentiation as mediated by scale-invariant adaptation, are also generic and have been observed in other systems including cortex^38^,^61^. Thus, our results provide a general mechanism by which SK channels can optimize neural responses to natural stimuli through temporal decorrelation/whitening, which in turn optimizes behavioural responses by making them best tuned to stimuli that occur most frequently in the natural environment. Optimized coding and perception of natural stimuli mediated by SK channels is thus likely to be a universal feature of sensory processing that is shared amongst systems and species.

## Methods

### Animals

The weakly electric fish *Apteronotus leptorhynchus* was used exclusively in this study. Animals were purchased from tropical fish suppliers and were acclimated to laboratory conditions according to published guidelines^62^. All procedures were approved by McGill University's animal care committee.

### Surgery

A total of 0.1–0.5 mg of tubocurarine (Sigma) was injected intramuscularly in order to immobilize the fish for experiments. The fish was respirated through a mouth tube at a flow rate of ∼10 ml min^−1^ when placed in the recording tank. To stabilize the head during recording, a metal post was glued to the exposed area of the skull. A small hole of ∼2 mm^2^ was drilled over the caudal lobe of the cerebellum above the ELL in order to gain access to the pyramidal neurons.

### Electrophysiology

We used well-established techniques to make extracellular recordings with Woods metal electrodes from pyramidal cells within the LS of the ELL^28^. We used CED 1401-plus hardware and Spike II software to record the resulting signal with resolution 0.1 ms.

### Pharmacology

The composition of the vehicle/control saline is as follows (all chemicals were obtained from Sigma): 111 mM NaCl, 2 mM KCl, 2 mM CaCl_2_, 1 mM MgSO_4_, 1 mM NaHCO_3_ and 0.5 mM NaH_2_PO_4_. The pH of the saline solution was 6.8. Glutamate (Sigma), UCL-1684 Ditrifluoroacetate hydrate (Sigma) and 1-EBIO 1-Ethyl-2-benzimidazolinone (Sigma) were dissolved in saline for application as before^43^. Drug application electrodes were two-barrel KG-33 glass micropipettes (OD 1.5 mm, ID=0.86 mm, A-M Systems) pulled by a vertical micropipette puller (Stoelting Co.) to a fine tip and subsequently broken to attain a tip diameter of ∼10 μm. The two barrels were used for separate application of either UCL-1684 (100 μM) or 1-EBIO (2.5 mM) and glutamate (1 mM). During recordings, we first used excitatory responses to glutamate application via PicoSpritzer to confirm that we were within proximity of the pyramidal neuron we were recording from as done previously^42^. UCL-1684 and 1-EBIO were then applied as done previously^43^.

### Behaviour

Animals were immobilized and set up in the recording tank similarly to the method described above. However, both ELLs were exposed and two glass micropipettes loaded with saline control solution, UCL-1684 (100 μM), or 1-EBIO (2.5 mM) solution were inserted into the LS segment using previously established techniques^42^,^44^. Simultaneous bilateral injection of either saline, UCL-1684, or 1-EBIO into the LS region of the ELL molecular were delivered via a PicoSpritzer (pressure=10 psi, pulse duration=140 ms). Sinusoidal waveforms with frequency of 4 Hz below the animal's baseline EOD frequency and with intensity of 2 mV cm^−1^ with duration of 50 s were presented. Previous studies have shown that such stimuli will reliably elicit a jamming avoidance response and/or transient EOD frequency excursion (that is, chirp) responses in *A. leptorhynchus*^42^. The jamming avoidance response magnitude was defined as the maximum frequency elicited during stimulation minus the baseline (that is, without stimulation) value and was used as a positive control to confirm that UCL-1684 had an effect. Envelope stimuli were then subsequently played and saline or UCL injected two or three times before each stimulus presentation. Behavioural sensitivity was measured as the ratio between the amplitude of the envelope stimulus as extracted by the dipole, and the response, which was quantified by the average extracted change in EOD frequency of the fish over the course of the stimulus. The phase relationship was quantified by determining the difference between the phase at which the maximum peak of the envelope stimulus occurred and the phase at which the maximum peak of the average extracted change in EOD frequency. *α*_behaviour_ was obtained by fitting a power law to the behavioural sensitivity as a function of frequency.

### Stimulation

The EOD of *A. leptorhynchus* is neurogenic, and therefore is not affected by injection of curare. All stimuli consisted of AMs of the animal's own EOD and were produced by triggering at the zero crossing of each EOD cycle as done previously^63^. This allowed the train of sinusoid stimuli to be synchronized to the animal's discharge and depending on the polarity, either added or subtracted from the animal's own discharge. The modulated waveform was subsequently multiplied (MT3 multiplier; Tucker Davis Technologies) and the resulting signal was isolated from ground (A395 linear stimulus isolator; World Precision Instruments). The signal was then delivered through a pair of chloridized silver wire electrodes placed ∼15 cm on either side of the recording tank perpendicular to the fish. The stimulus intensity was adjusted to give rise to changes in EOD amplitude that was ∼20% of the baseline level as in previous studies^63^ that were measured using a small dipole placed close to the animal's skin. The stimuli consisted of two noisy AM waveforms with frequency contents 5–15 and 60–80 Hz, whose envelopes were modulated, sinusoidally with frequencies ranging from 0.05 to 1 Hz (ref. 15) or in a stepwise fashion at frequencies 0.05, 0.1, 0.25, 0.5, 1, 2 and 4 Hz for 5–15 Hz and 0.05, 0.1, 0.25, 0.5, 1, 2, 4, 8 and 16 Hz for 60–80 Hz (note that the step duration is then half of the stimulus period). Stimuli also consisted of envelope stimulus waveforms obtained under natural conditions^18^ as well as noisy waveforms whose power spectrum decayed as a power law with exponent *α*_stim_=−0.8 and whose phase varied uniformly. The slope of the spike triggered average computed in response to the noisy AM waveform was used to assign each cell as either ON of OFF-type as done previously^64^.

### Fractional differentiation model

Fractional differentiation^65^ can be described simply as the differentiation operation, d^*α*^/d*t*^*α*^, in which the order of differentiation, is a non-integer number. In the frequency domain, fractional differentiation of order *α* corresponds to filtering by a transfer function *H(f)* given by


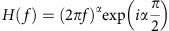


The gain *G(f)* and phase *φ(f)* of the model can then be written as


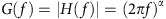



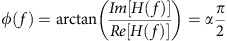


where *Im*[*H*(*f*)] and *Re*[*H*(*f*)] are the imaginary and real parts, respectively. We fitted a fractional differentiation model to our data using the Grunwald–Letnikov definition, which was adapted to use a vectorization method to pass signals through a spectrum of fractional derivative values between 0 and 1 from which we obtained *α*_neuron_^65^.

### Matching response sensitivity to stimulus statistics in order to ensure temporal decorrelation

Linear response theory^66^ posits that the response power spectrum *P*_*rr*_*(f)* is related to the gain *G(f)* and the stimulus power spectrum *P*_*ss*_*(f)* by the following equation:


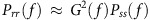


Thus, if the stimulus power spectrum decays as a power law with exponent *α*_stim_ and if the neural gain increases as a power law with exponent *α*_neuron_, then we have


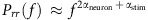


The response power spectrum will then be independent of frequency *f* if 2*α*_neuron_*+α*_stim_=0 or, equivalently, if


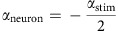


### Relationship between neural tuning and behaviour

We assume that the neural tuning exponent *α*_neuron_ and the behavioural exponent *α*_behavioural_ are related by


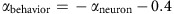


We then have


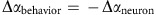


where Δ*α* is the change in exponent *α* resulting from pharmacological manipulation of SK channels.

### Neuron model

To model the responses of the pyramidal neurons to the stimuli used in this study, we implemented a leaky integrate-and-fire model with power-law adaptation


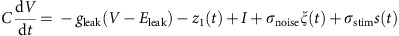


where *C* is the membrane capacitance, *g*_leak_ is the leak conductance, *E*_leak_ is the leak reversal potential, *I* is a constant bias current, *ξ(t)* is gaussian white noise with zero mean and s.d. unity, *σ*_noise_ is the noise intensity, *s(t)* is the stimulus which was taken to have the same statistics as for the data, *σ*_stim_ is the stimulus intensity, *V* is the membrane potential and *z*_*1*_*(t)* is the adaptation current. Each time the membrane potential reaches the threshold *θ*, it is reset to *V*_reset_ and an action potential is said to have occurred at that time. The adaptation current is then incremented.

We approximated the power law adaptation using *N* variables *z*_1_…*z*_*N*_ that obeyed the following system of differential equations^37^:


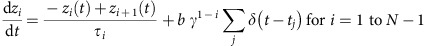



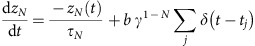



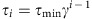


where *t*_*j*_ are the spikes times, *δ(t)* is the delta function, and *b* and *γ* are constants that determine the strength and power law exponent *α*_neuron_ of the neural sensitivity, respectively. The model was simulated using an Euler–Maruyama integration with timestep d*t*=0.025 ms. We used parameter values *C*=1 μF cm^−2^, *g*_leak_=0.36 μS cm^−2^, *E*_leak_=−70 mV, *I*= μA cm^−2^, *σ*_noise_= μA cm^−2^, *N*=40, *b*=0.2, *γ*=1.1253, *θ*=−50 mV, *V*_reset_=−70 mV and *C*=1 μF cm^−2^. For these parameter values, we obtained *α*_model_=0.4.

### Data quantification

We used several methods in order to quantify our experimental data. Correlation time was measured as the duration of time it took to decay to 5% of maximum autocorrelation value. White index was measured by taking the normalized area under the power spectrum curve using a trapezoidal method and dividing by the maximum normalized area to achieve a value between 0 and 1. The match between behaviour and natural stimulus statistics was obtained as 1−|α_stim_−α_behaviour_| and thus is maximum when the two power-law exponents match. This method was used in order to quantify the optimality of the animal's behaviour during the pharmacology experiments. For step envelope stimuli, we constructed PSTHs by averaging over each step onset and offset and typically used 50 bins for a given step duration.

## Additional information

**How to cite this article:** Huang, C. G. *et al.* Temporal decorrelation by SK channels enables efficient neural coding and perception of natural stimuli. *Nat. Commun.* 7:11353 doi: 10.1038/ncomms11353 (2016).

## Supplementary Material

Supplementary Information Supplementary Figures 1-3
