# Supplementary material for: Temporal decorrelation by SK channels enables efficient neural coding and perception of natural stimuli
Source: Nat Commun. 2016 Apr 18;7:11353. doi: 10.1038/ncomms11353 (PMC4837484; doi:10.1038/ncomms11353)
Supplement: Supplementary Information — Supplementary Figures 1-3 [file ncomms11353-s1.pdf]

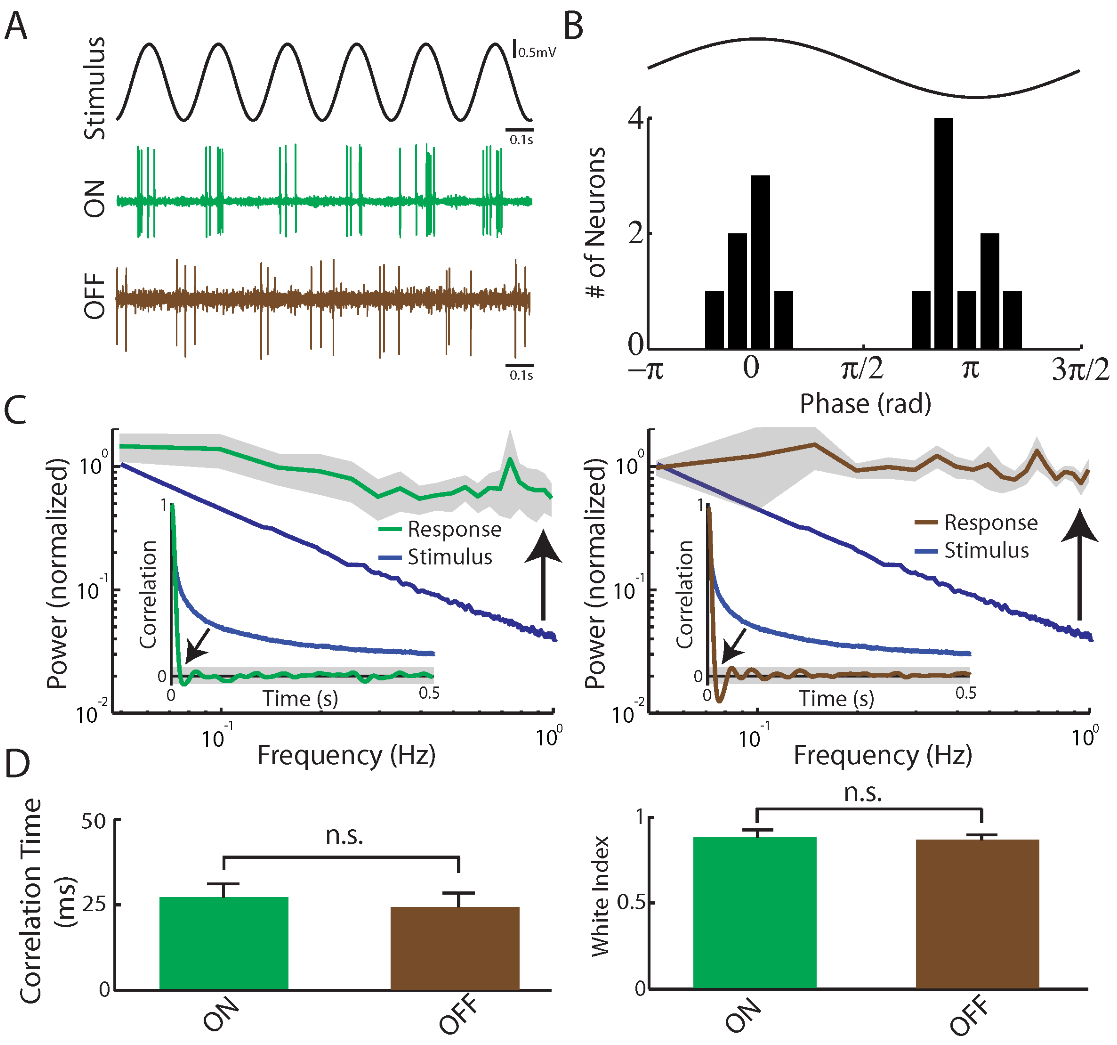

Supplementary Figure 1: ON and OFF-type ELL pyramidal cells display similar responses to second order attributes of natural electrosensory stimuli. **A:** Example responses of example ON-type (green) and OFF-type (brown) ELL pyramidal cells to a 4 Hz sinusoidal AM (black). **B:** Distribution of stimulus phase for which ELL pyramidal cells in our dataset fired preferentially. The distribution is clearly bimodal (Hartigan's dip test, $p=0.0167$) with ON-type cells firing preferentially near the maximum of the stimulus (phase 0) and OFF-type cells firing preferentially near the minimum (phase π). **C:** The population-averaged response power spectrum (green) for ON (left) and (brown) OFF (right) type cells was relatively constant as compared to that of the envelope stimulus (blue). Insets: The population-averaged response autocorrelation function (green) for ON (left) and (brown) OFF (right) type cells decayed to zero much faster than that of the stimulus (blue). **D:** Population-averaged correlation times (left) and white index (right) for ON (green) and OFF (brown) type cells. No significant differences were observed between correlation time (Wilcoxon rank-sum test, $p>0.05$, n.s., $N=14$) or white index values (Wilcoxon rank-sum test, $p>0.05$, n.s., $N=14$).

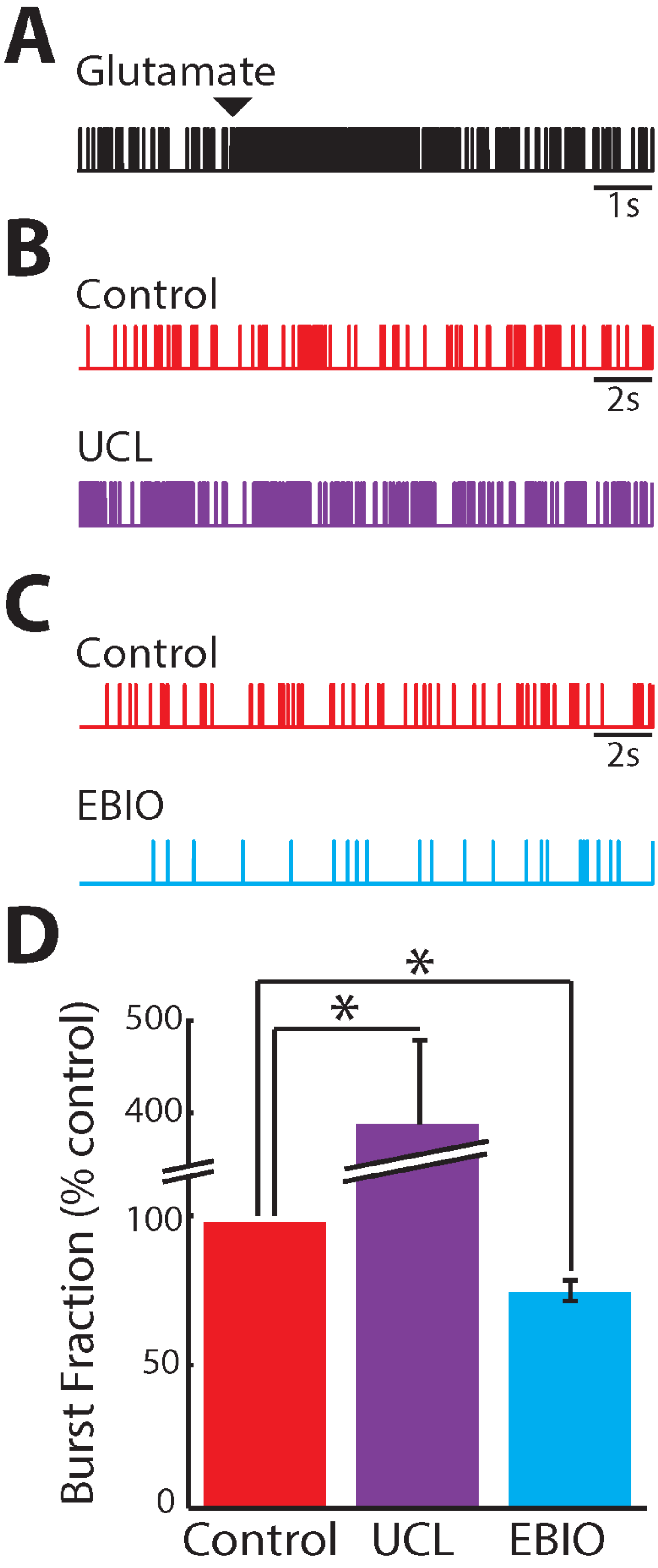

Supplementary Figure 2: *UCL and EBIO application have opposite effects on pyramidal neuron baseline activity.* **A)** Glutamate ejection causes rapid increases in pyramidal neuron firing rate, indicating that the pharmacology electrode is close to the neuron from which we are recording. **B)** Baseline activity under control (top) and after UCL application (bottom) from a typical pyramidal neuron. **C)** Same as B for EBIO application. **D)** Population-averaged burst fractions under baseline (control) and after UCL and EBIO application, respectively. Burst fraction was significantly different between control and UCL (Wilcoxon rank-sum test, $p<0.05$, N=6) and between control and EBIO (Wilcoxon rank-sum test, $p<0.05$, N=6).

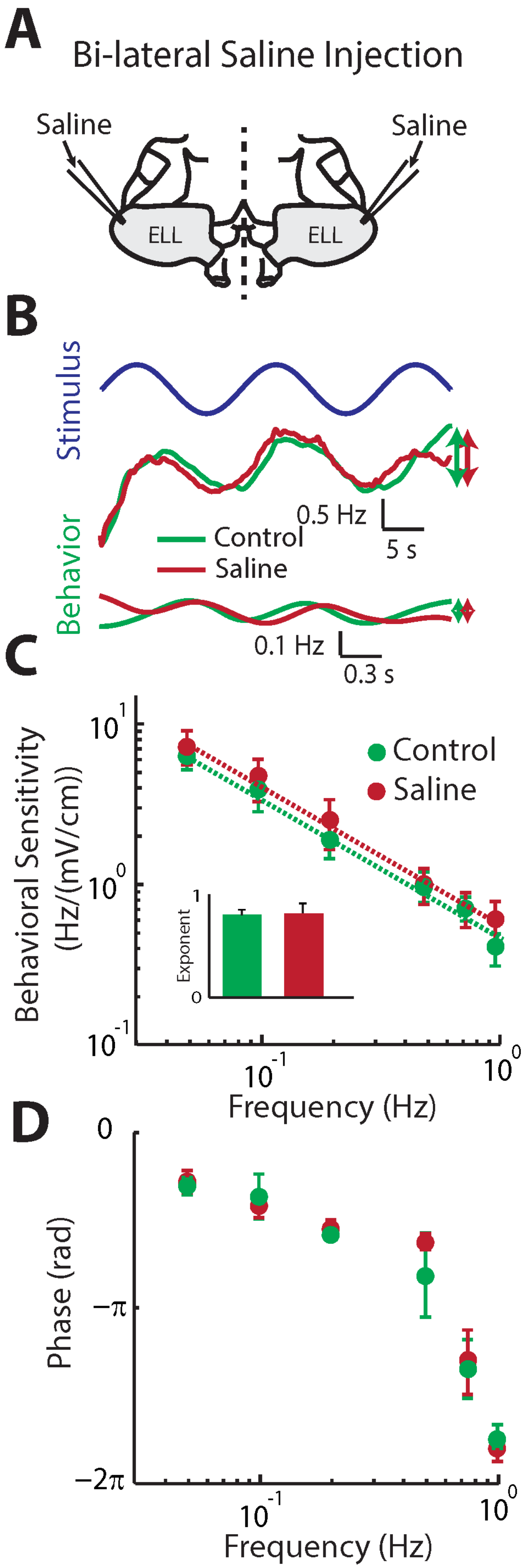

Supplementary Figure 3: *Saline injection does not significantly alter behavioral responses to envelope stimuli.* **A)** Schematic showing the bilateral saline injection. **B)** *Top:* Low (*left)* and high (*right*) frequency envelope stimuli. *Bottom:* Corresponding behavioral responses before (green) and after (red) saline injection. **C)** Population-averaged behavioral sensitivity before (green) and after (red) saline injection. The dashed lines show the best power law fits to the data. Inset: Population-averaged power law exponents for before (green) and after saline injection (red) (N=3). **D)** Population-averaged phase lag before (green) and after (red) saline injection (N=3).
